# A Systematic Review on GLP-1 Receptor Agonists in Reproductive Health: Integrating IVF Data, Ovarian Physiology and Molecular Mechanisms

**DOI:** 10.3390/ijms27020759

**Published:** 2026-01-12

**Authors:** Charalampos Voros, Fotios Chatzinikolaou, Ioannis Papapanagiotou, Spyridon Polykalas, Despoina Mavrogianni, Aristotelis-Marios Koulakmanidis, Diamantis Athanasiou, Vasiliki Kanaka, Kyriakos Bananis, Antonia Athanasiou, Aikaterini Athanasiou, Georgios Papadimas, Charalampos Tsimpoukelis, Dimitrios Vaitsis, Athanasios Karpouzos, Maria Anastasia Daskalaki, Nikolaos Kanakas, Marianna Theodora, Nikolaos Thomakos, Panagiotis Antsaklis, Dimitrios Loutradis, Georgios Daskalakis

**Affiliations:** 1 1st Department of Obstetrics and Gynecology, ‘Alexandra’ General Hospital, National and Kapodistrian University of Athens, 80 Vasilissis Sofias Avenue, 11528 Athens, Greece; 2 Laboratory of Forensic Medicine and Toxicology, School of Medicine, Aristotle University of Thessaloniki, 54124 Athens, Greece; loutradi@otenet.gr; 3 Athens Medical School, National and Kapodistrian University of Athens, 15772 Athens, Greece; 4 King’s College Hospitals NHS Foundation Trust, London SE5 9RS, UK; 5 IVF Athens Reproduction Center, 15123 Maroussi, Greece; 6 School of Medicine, European University Cyprus, Nicosia 2404, Cyprus; 7 Fertility Institute-Assisted Reproduction Unit, Paster 15, 11528 Athens, Greece

**Keywords:** infertility, oxidative stress, *SIRT1*, Nrf2, ferroptosis, ovarian ageing, antioxidants, IVF, reproductive endocrinology, molecular research

## Abstract

Women of reproductive age, especially those with polycystic ovarian syndrome (PCOS), often use glucagon-like peptide-1 receptor agonists (GLP-1RAs) to improve their metabolic functions. A growing body of evidence suggests that GLP-1R signaling may directly affect ovarian physiology, influencing granulosa cell proliferation, survival pathways, and steroidogenic production, in addition to its systemic metabolic effects. Nonetheless, there is a limited comprehension of the molecular mechanisms that regulate these activities and their correlation with menstrual function, reproductive potential, and assisted reproduction. This comprehensive review focuses on ovarian biology, granulosa cell signaling networks, steroidogenesis, and translational fertility outcomes, integrating clinical, in vivo, and in vitro information to elucidate the effects of GLP-1 receptor agonists on reproductive health. We conducted a thorough search of PubMed, Scopus, and Web of Science for randomized trials, prospective studies, animal models, and cellular experiments evaluating the effects of GLP-1RA on reproductive or ovarian outcomes, in accordance with PRISMA criteria. The retrieved data included metabolic changes, androgen levels, monthly regularity, ovarian structure, granulosa cell growth and death, *FOXO1* signaling, FSH-cAMP-BMP pathway activity, and fertility or IVF results. Clinical trials shown that GLP-1 receptor agonists improve menstrual regularity, decrease body weight and central adiposity, increase sex hormone-binding globulin levels, and lower free testosterone in overweight and obese women with PCOS. Liraglutide, when combined with metformin, significantly improved IVF pregnancy rates, whereas exenatide increased natural conception rates. Mechanistic studies demonstrate that GLP-1R activation affects *FOXO1* phosphorylation, hence promoting granulosa cell proliferation and anti-apoptotic processes. Incretin signaling altered steroidogenesis by reducing the levels of StAR, P450scc, and 3β-HSD, so inhibiting FSH-induced progesterone synthesis, while simultaneously enhancing BMP-Smad signaling. Animal studies demonstrated both beneficial (enhanced follicular growth, anti-apoptotic effects) and detrimental results (oxidative stress, granulosa cell death, uterine inflammation), indicating a context- and dose-dependent response. GLP-1 receptor agonists influence female reproductive biology by altering overall physiological processes and specifically impacting the ovaries via *FOXO1* regulation, steroidogenic enzyme expression, and BMP-mediated FSH signaling. Preliminary clinical data indicate improved reproductive function in PCOS, as seen by increased pregnancy rates in both natural and IVF cycles; nevertheless, animal studies reveal a potential risk of ovarian and endometrial damage. These results highlight the need for controlled human research to clarify reproductive safety, molecular pathways, and optimum therapy timing, particularly in non-PCOS patients and IVF settings.

## 1. Introduction

### 1.1. GLP-1 Physiology and Systemic Metabolic Actions

Glucagon-like peptide-1 (GLP-1) is an incretin hormone synthesised by enteroendocrine L-cells in the distal ileum and colon in response to food consumption, particularly carbs and fats. Upon its release, GLP-1 regulates nutrition utilisation, energy storage, and metabolic stability via several endocrine and paracrine mechanisms [1]. Dipeptidyl peptidase-4 (DPP-4) rapidly degrades the peptide, resulting in a brief physiological half-life of around 1–2 min. This issue has been addressed by pharmacological GLP-1 receptor agonists (GLP-1RAs) with an extended duration of action [2]. The GLP-1 receptor (GLP-1R) is a class B G protein-coupled receptor (GPCR) mostly associated with Gαs proteins. It activates adenylate cyclase and increases intracellular cyclic AMP (cAMP) levels [3]. cAMP augments insulin gene transcription, insulin granule exocytosis, and β-cell survival in pancreatic β-cells by activating protein kinase A (PKA) and exchange protein activated by cAMP (EPAC) via anti-apoptotic pathways (e.g., PI3K/AKT). GLP-1 inhibits α-cells from secreting glucagon, delays gastric emptying, and modifies the hypothalamic satiety centres via both vagal afferent and central pathways [4]. GLP-1 signalling alters the NF-κB, c-Jun N-terminal kinase (JNK), and mitogen-activated protein kinase (MAPK) pathways, producing effects that extend beyond glucose regulation, including the reduction in inflammation, oxidative stress, and atherosclerosis.

Researchers have identified GLP-1R expression in adipose tissue, cardiomyocytes, endothelial cells, the renal system, and the central nervous system. This indicates that it is not only located in metabolic organs [5]. These extrapancreatic actions enhance insulin sensitivity, improve lipid metabolism, reduce systemic inflammation, and regulate autonomic output. Activation of GLP-1R in adipocytes inhibits the activity of pro-inflammatory cytokines such as TNF-α and IL-6, while initiating lipolysis, browning, and the secretion of adiponectin [6]. Liraglutide, semaglutide, dulaglutide, and exenatide are pharmacological agents classified as GLP-1RAs. They are engineered to withstand DPP-4 degradation and possess half-lives ranging from hours to days. Their therapeutic outcomes include substantial weight reduction, decreased visceral fat, improved blood glucose levels, reduced fatty liver disease, and enhanced cardiovascular and metabolic health. The circuits governing reproductive physiology are intricately connected to these systemic improvements [7]. Enhanced insulin sensitivity mitigates ovarian androgen surplus induced by excessive insulin, whereas reduced inflammation in adipocytes restores the normal signalling of the hypothalamic-pituitary-ovarian axis (HPO). GLP-1 traverses the blood–brain barrier, influencing hypothalamus nuclei that regulate the pulsatility of gonadotropin-releasing hormone (GnRH) [8]. GLP-1 may indirectly support ovulatory function by improving metabolic signals that link peripheral nutrition status with reproductive preparedness.

### 1.2. Obesity, PCOS, and Reproductive Dysfunction

Obesity significantly impacts the female reproductive system by influencing the HPO axis via metabolic, endocrine, and inflammatory mechanisms. Individuals who are overweight or obese have persistent low-grade inflammation and insulin resistance, resulting in elevated insulin levels [9]. Elevated concentrations of insulin and luteinizing hormone (LH) synergistically enhance the activity of CYP17A1 in ovarian theca cells, resulting in excessive androgen synthesis. Hyperandrogenism impedes anovulation, granulosa cell development, and folliculogenesis [10]. Insulin inhibits the liver’s production of sex hormone-binding globulin (SHBG), exacerbating the hyperandrogenic condition by increasing the concentration of free androgens in the bloodstream. Adipose tissue functions as an active endocrine organ, synthesising adipokines including leptin, adiponectin, resistin, and pro-inflammatory cytokines such as TNF-α, IL-1β, and IL-6 [11]. These chemicals may affect reproductive function. Insufficient adiponectin levels diminish insulin-sensitizing signals and exacerbate metabolic issues. Alterations in leptin signalling, conversely, affect the dynamics of GnRH pulses and the functioning of LH/FSH. Visceral fat exacerbates these effects by inducing oxidative stress, impairing mitochondrial function, and altering the expression of steroidogenic enzymes in the ovary.

PCOS exemplifies the connection between metabolism and reproduction. Between 8% and 13% of women of reproductive age are affected by it. PCOS is characterised by chronic anovulation, elevated androgen levels in the bloodstream or body, and a polycystic ovarian morphology [12]. It is often associated with insulin resistance and metabolic syndrome. Impairments in granulosa cell signalling, such as deregulation of the PI3K/AKT pathway, elevated concentrations of anti-Müllerian hormone (AMH), and aberrant FSH-mediated aromatase activity, lead to follicular stoppage in the early antral stage [13]. In PCOS, the ovarian environment demonstrates increased oxidative stress, mitochondrial impairment, and modifications in autophagic and apoptotic pathways. All of these factors diminish the efficacy of oocytes in performing their function. These alterations in metabolism and hormones extend beyond the ovary [14]. Obesity and PCOS alter the expression of progesterone receptors, integrin profiles, vascular remodelling, and local inflammatory signalling, rendering the endometrium less susceptible. Consequently, both natural conception and assisted reproduction provide worse outcomes. Obese women undertaking IVF often need more gonadotropin dosages, yield a diminished number of mature oocytes, generate embryos of worse quality, and encounter decreased implantation and live birth rates. Long-term metabolic inflammation and inadequate luteal function have been associated with an elevated risk of miscarriage [15].

Weight reduction is a crucial factor in assisting obese individuals and those with PCOS in achieving pregnancy. Reducing body weight by as little as 5–10% might enhance menstrual regularity, boost ovulation frequency, and elevate the incidence of clinical pregnancies. However, it remains challenging to lose weight and maintain that loss, underscoring the need of discovering methods to enhance metabolism [16]. GLP-1 receptor agonists effectively mitigate the metabolic factors contributing to reproductive dysfunction by enhancing insulin efficacy, reducing inflammation in adipocytes, and specifically targeting visceral fat. The modifications to the whole system amplify and increase the likelihood of direct effects on the ovaries resulting from GLP-1R signalling [17].

### 1.3. GLP-1 Signaling in the Ovary

Although GLP-1R has traditionally been studied in pancreatic, gastrointestinal, and neurological tissues, recent findings reveal the existence of GLP-1R in the ovary, particularly in the GCs of developing follicles. This discovery presents a physiologic basis for the potential direct effect of GLP-1Ras on the ovaries, irrespective of enhancements in systemic metabolism [18]. The presence of GLP-1R in the ovaries suggests that incretin signalling may affect essential pathways regulating follicular survival, steroidogenesis, and oocyte maturation. GLP-1R is a type B G protein-coupled receptor that primarily induces the accumulation of cAMP inside cells by activating the Gαs-adenylate cyclase pathway. cAMP serves as a crucial second messenger in granulosa cells [19]. It governs the production of steroid hormones, the expression of aromatase, and differentiation mediated by FSH. GLP-1R signalling may modulate essential activities like GC proliferation, apoptosis, and gonadotropin responsiveness via the activation of PKA and exchange protein directly activated by cAMP (EPAC) downstream.

Experimental findings indicate that a primary downstream target of GLP-1R signalling is *FOXO1*, an essential transcription factor crucial for GC apoptosis and cell cycle control. In murine models of PCOS, the injection of GLP-1RA enhances *FOXO1* phosphorylation, leading to its exclusion from the nucleus and ultimately diminishing its pro-apoptotic function [18]. This process enhances the viability of granulosa cells, perhaps facilitating follicular maturation. The heightened death of granulosa cells often seen in PCOS, associated with oxidative stress, hyperandrogenism, and metabolic dysregulation, may be alleviated by these anti-apoptotic actions [20]. The BMP-Smad pathway, an essential regulator of ovarian steroidogenesis, concurrently interacts with GLP-1 signalling. GLP-1 inhibits FSH-induced progesterone production in in vitro models by reducing the expression of steroidogenic enzymes, such as StAR, P450scc, and 3β-HSD. Augmented BMP-Smad1/5/8 activation inhibits progesterone production, hence contributing to this suppression. The results suggest that incretin signalling may precisely regulate steroidogenic production via the interaction of FSH-cAMP and BMP-Smad pathways, leading to a context-dependent alteration of follicular endocrine function [21].

The effect of GLP-1RAs on the ovaries is markedly affected by the physiological setting, treatment period, and dose given. Certain studies indicate that liraglutide may be beneficial by accelerating the proliferation of granulosa cells, inhibiting apoptosis, and enhancing follicular development. However, further research, particularly with animals given high doses of liraglutide, indicates potential adverse effects. These factors include heightened oxidative stress, mitochondrial dysfunction, granulosa cell death, and modified ovarian shape, all indicating that excessive or extended exposure to GLP-1RA may disturb ovarian homeostasis.

### 1.4. Rationale for GLP-1RA Use in Reproductive Medicine

GLP-1RAs have garnered interest in reproductive medicine for their ability to mitigate the interrelated metabolic, endocrine, and ovarian dysfunctions that contribute to various forms of female infertility, particularly those associated with obesity and PCOS [22]. GLP-1RAs demonstrate several advantages, including substantial weight reduction, enhanced insulin sensitivity, less systemic inflammation, and the reinstatement of metabolic indicators associated with ovarian dysfunction. Enhancing metabolism using GLP-1 receptor agonists offers a solid theoretical basis for enhancing reproductive outcomes, given that obesity and insulin resistance affect follicular maturation, ovulation, egg quality, and endometrial receptivity [23].

Additionally, GLP-1 receptor agonists seem to provide reproductive advantages with systemic metabolic enhancement. Clinical investigations indicate that GLP-1 receptor agonists assist overweight or obese women with PCOS in achieving more regular menstrual cycles, increased spontaneous ovulation, reduced excess testosterone levels, and normalised gonadotropin-independent ovarian function [22,24]. These enhancements indicate intragonadal mechanisms that might enhance systemic effects. Preliminary findings indicate that GLP-1RA medication combined with metformin enhances IVF-related outcomes, including the pregnancy rate per embryo transfer, despite equivalent weight reduction across treatment groups. The results suggest that GLP-1 receptor agonists may influence endometrial or follicular biology in ways not exclusively linked to variations in body adiposity [25].

Mechanistic evidence supports this notion. The presence of GLP-1 receptors in granulosa cells indicates that GLP-1 receptor agonists might directly affect ovarian mechanisms governing steroidogenesis, follicular maturation, and granulosa cell survival. In PCOS models, GLP-1R activation has been shown to alter *FOXO1* phosphorylation, diminish apoptosis, and promote cellular proliferation [18]. Conversely, investigations demonstrate that supraphysiological dosages of GLP-1 receptor agonists may cause granulosa cell degeneration, increase oxidative stress, and alter the expression of steroidogenic enzymes. This duality illustrates the complexity of GLP-1RA activity in reproductive organs and underscores the significance of dosage, treatment duration, and physiological environment [26].

There are considerable apprehensions about the safety and effectiveness of GLP-1RAs during ovarian stimulation, early pregnancy, and ART, especially due to their rising use among women of reproductive age, frequently commenced for weight reduction rather than diabetes treatment [27]. Notwithstanding encouraging first results, there is a deficiency of data about their effects on egg quality, embryo development, endometrial receptivity, or peri-implantation physiology in women devoid of PCOS. The lack of comprehensive mechanistic research in human ovarian tissue hinders the distinction between direct and indirect effects.

In reproductive medicine, GLP-1 receptor agonists were initially conceptualised in early integrative and narrative studies primarily as metabolic agents that indirectly improved ovulatory function and fertility by facilitating weight loss, insulin sensitisation, and systemic endocrine normalisation in obese women and patients with PCOS. This significant research established the foundation for the metabolic-reproductive axis and showed that obesity and insulin resistance are primary factors contributing to ovarian dysfunction.

However, mounting experimental and translational evidence suggests that GLP-1 receptor signalling may have direct intra-ovarian effects on granulosa cell viability, steroidogenic enzyme expression, and gonadotropin responsiveness. This systematic review enhances early integrative perspectives by offering a structured synthesis of clinical IVF results with mechanistic in vitro and in vivo findings. It also illustrates the evolution of the science from examining metabolic connections to investigating molecular and ovarian-specific processes.

This systematic review aims to thoroughly assess the clinical, translational, and molecular effects of glucagon-like peptide-1 receptor agonists (GLP-1RAs) on reproduction. This review seeks to consolidate information on three main enquiries: (i) the effectiveness of GLP-1 receptor agonists in improving reproductive and IVF outcomes in women of reproductive age, especially those with obesity or polycystic ovary syndrome; (ii) the influence of GLP-1 signalling on ovarian physiology at cellular and molecular levels, including granulosa cell survival, steroidogenesis, and gonadotropin responsiveness; and (iii) the safety implications derived from findings in experimental animals and in vitro studies.

We comprehensively included randomised clinical trials, observational human studies, animal models, and in vitro studies that assessed the effects of GLP-1 receptor agonist exposure on reproductive, ovarian, hormonal, or fertility-related outcomes. This comprehensive method was designed to clarify the therapeutic potential and biological constraints of GLP-1 receptor agonists in female reproductive health, while synthesising clinical data with molecular insights.

## 2. Materials and Methods

Our systematic review was carried out in accordance with PRISMA 2020 guidelines. The PROSPERO registration number is CRD420251269164. A comprehensive search of PubMed, Scopus, and Web of Science was carried out between the database’s creation and December 2025. GLP-1 receptor agonists and reproductive biology-related MeSH terms and free-text keywords include “GLP-1 receptor agonist,” “liraglutide,” “exenatide,” “semaglutide,” “incretin,” “ovary,” “granulosa cells,” “steroidogenesis,” “*FOXO1*,” “BMP,” “PCOS,” “female infertility,” “pregnancy,” and “IVF.” The reference lists of relevant articles were manually screened to identify any additional relevant studies. The complete search strategy is summarized in Table 1.

Following the search approach outlined in Table 1, papers were evaluated based on predetermined inclusion and exclusion criteria. Eligible studies were randomised controlled trials, prospective or retrospective human observational studies, experimental animal models, and in vitro studies assessing the impact of GLP-1 receptor agonists on ovarian or reproductive outcomes. Studies required to assess key outcomes such as menstrual cyclicity, ovulation, metabolic and androgen profiles, ovarian morphology, granulosa cell function, steroidogenic enzyme expression, oxidative stress, apoptosis, natural conception, and IVF-related factors. The populations of interest included women with PCOS or obesity, women of reproductive age without PCOS, animal models administered GLP-1 receptor agonists, and native or cultured granulosa cells. The treatments included all GLP-1 receptor agonists (e.g., liraglutide, exenatide, semaglutide, and dulaglutide) and GLP-1 signalling pathways evaluated at the molecular level. We excluded reviews, meta-analyses, editorials, conference abstracts lacking complete data, case reports, studies unrelated to ovarian or reproductive outcomes, publications in languages other than English, and studies where the specific effect of GLP-1RA could not be isolated due to combined interventions.

### Study Selection

**Figure 1:**
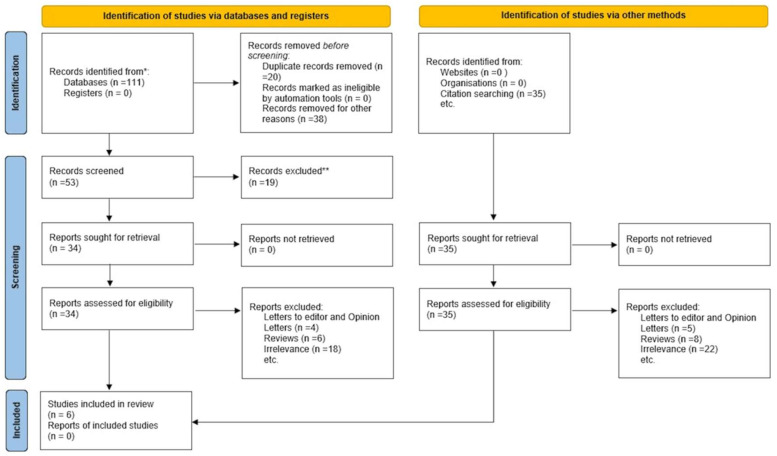
Prisma 2020 flow diagram for systematic reviews, which included searches of databases, registers and other sources.

A total of 1284 records were identified in the database searches, and manual reference filtering uncovered an additional 7 records. Subsequent to the elimination of 312 duplicates, 979 titles and abstracts were examined. 942 of these were excluded due to their non-original nature (reviews, comments, or editorials), lack of discussion on ovarian or reproductive outcomes, or failure to use GLP-1 receptor agonists. We assessed the eligibility of the remaining 37 full-text papers. Twenty-eight studies were rejected for failing to identify the effects of GLP-1 signalling, using DPP-4 inhibitors without analysing GLP-1 receptor agonists, including inappropriate populations, or lacking relevant reproductive outcomes. Nine research three randomised clinical trials, one human observational study, three in vivo animal studies, and two in vitro granulosa cell investigations satisfied the inclusion criteria. Figure 1, the PRISMA flow diagram, illustrates the comprehensive process of selecting research.

## 3. Results

The included studies exhibited significant variability in their designs, demographics, intervention types, and reproductive outcomes. Table 2 enumerates the principal components of all eligible clinical trials, in vitro granulosa cell tests, and in vivo animal research. Three randomised controlled studies investigated the impact of liraglutide or exenatide on menstrual cyclicity, metabolic improvement, ovulation, natural conception, and IVF outcomes in women with PCOS or obesity. A human observational research evaluated ovarian and bleeding parameters without reproductive intervention. Three animal studies examined the impact of liraglutide on ovarian histology, granulosa cell apoptosis, steroidogenesis, and sex hormone concentrations. Two mechanistic in vitro investigations examining the influence of GLP-1 on *FOXO1*, cAMP, BMP–Smad signalling, and steroidogenic enzymes (*StAR*, P450scc, 3β-HSD) provided significant insights into the functionality of granulosa cells. The studies collectively covered a thorough translational continuum, from molecular signalling to clinical pregnancy, enabling an integrated synthesis of GLP-1RA effects on ovarian physiology and fertility, despite considerable variations in intervention duration (ranging from days to 24 weeks), dosage regimens, and assessed reproductive outcomes. A thorough overview of study designs, participant characteristics, GLP-1RA treatment plans, and reproductive outcomes is provided in Table 2.

Table 2 summarises the design, demographic, intervention methods, and reproductive endpoints of the research included in this review. The three randomised clinical studies investigating GLP-1 receptor agonists in overweight or obese women with PCOS Salamun et al., Liu et al., and Nylander et al., constitute the most significant human evidence [28,29,30]. The trials showed that GLP-1RA medication enhanced metabolic indices, menstrual regularity, androgen levels, and both spontaneous and IVF-related pregnancy outcomes, despite variations in sample size, dose, and treatment duration. The beneficial effects were evident despite comparable weight loss in the intervention groups, suggesting that GLP-1RAs may provide reproductive advantages beyond basic adiposity reduction.

The integrated human observational research, by evaluating baseline ovarian and hormonal parameters across diverse populations without targeted pharmaceutical intervention, underscores the therapeutic significance of GLP-1RA effects on ovarian physiology. These human studies together provide significant new insights into the possible effects of GLP-1 receptor agonists on endometrial receptivity, androgen excess, and ovulatory function. The in vivo animal investigations clarify the processes by showing that liraglutide causes significant changes in ovarian histology, including granulosa-cell death, follicular atresia, and abnormalities in oxidative stress indicators. The data indicate that GLP-1RA exposure might have both beneficial and detrimental effects on the ovaries, contingent upon the dosage and duration of therapy. These investigations indicate alterations in the levels of reproductive hormones such as FSH, LH, oestrogen, progesterone, and testosterone. This signifies modifications in the endocrine system at both the ovarian and systemic levels.

The in vitro investigations provide extensive molecular insights into GLP-1-mediated intracellular signalling in granulosa cells, corroborating the in vivo observations. These results show that GLP-1 and GIP may influence essential regulatory pathways, including *FOXO1* phosphorylation, cAMP–PKA activation, BMP–Smad signalling, and the production of steroidogenic enzymes such as 3β-HSD, StAR, and P450scc. The mechanistic results indicate many interaction sites between GLP-1RA signalling and essential processes governing the survival, proliferation, apoptosis, and steroidogenesis of granulosa cells. Collectively, the findings included in Table 2 enhance our understanding of cellular function, systemic physiology, and clinical outcomes in individuals. This integrated approach highlights areas lacking sufficient evidence, including embryo development, endometrial receptivity, and reproductive safety in individuals without PCOS. It also aids in understanding how GLP-1 receptor agonists may influence women’s reproductive health, including alterations in metabolism or direct effects on the ovaries.

### 3.1. Clinical Outcomes

#### 3.1.1. IVF and Assisted Reproduction Outcomes

The forthcoming randomised pilot research by Salamun et al., currently the only clinical trial assessing IVF results in women undergoing preconception GLP-1RA medication, serves as the primary source of information about the impact of GLP-1RAs on assisted reproduction. This trial included administering metformin to obese women with PCOS, either as a monotherapy or in conjunction with low-dose liraglutide (1.2 mg/day), for a duration of 12 weeks. Subsequently, there was a discontinuation of medication and ovarian stimulation. The combo medication resulted in a markedly elevated clinical pregnancy rate per embryo transfer (85.7% vs. 28.6%), indicating a possibly independent influence of GLP-1 signalling on reproductive physiology beyond mere weight reduction, despite both groups experiencing equivalent weight loss. Numerous factors may have contributed to this remarkable transformation. Initially, GLP-1RA therapy significantly enhanced insulin sensitivity, reduced blood free testosterone levels, and increased SHBG levels. These alterations enhanced the endocrine milieu for folliculogenesis and prepared the endometrium. Mitigating hyperinsulinemia and androgen excess may enhance oocyte developmental competence and implantation potential, since both circumstances are recognised to compromise granulosa cell activity, oocyte maturation, and endometrial receptivity.

Secondly, the significant improvement in IVF pregnancy rates, despite similar weight patterns, clearly suggests that GLP-1RA medication may confer direct benefits to ovarian function. This theory aligns with mechanistic evidence from in vitro and animal studies, demonstrating that GLP-1 and GLP-1RAs regulated the expression of steroidogenic enzymes, reduced granulosa cell apoptosis, enhanced proliferative survival pathways, and affected *FOXO1* activity. These intrafollicular effects may facilitate optimal oocyte maturation, reduce oxidative stress, and improve mitochondrial function all critical criteria for IVF success. Third, there is increasing evidence that GLP-1 receptor agonists may potentially influence endometrial receptivity. Enhancements in inflammatory and metabolic indicators, such as decreased TNF-α, IL-6, and insulin resistance, may facilitate ideal endometrial gene expression profiles associated with implantation, including integrins, HOXA10, IGFBP-1, and angiogenic pathways. The unequal increase in implantation rates compared to weight fluctuations suggests potential impacts at the endometrial level, despite Salamun et al. not doing endometrial profiling.

It is essential to acknowledge that none of the other clinical studies explicitly evaluated IVF outcomes; still, their findings imply that GLP-1 receptor agonists may enhance the efficacy of assisted reproductive technology. Liu et al. and Nylander et al. demonstrated enhancements in menstrual cyclicity, ovarian morphology, elevated levels of SHBG, reduced levels of androgens, and metabolic stability [29,30]. All of them are upstream variables that influence ovarian response to stimulation and embryonic development. Nonetheless, some restrictions must be acknowledged. The only research investigating IVF had a restricted sample size and only included obese women with PCOS, making it difficult to generalise to other demographics. The timing, duration, and dosage of GLP-1 receptor agonists prior to IVF remain ambiguous, as do the sustained advantages for women without PCOS, those of normal weight, or those who exhibit poor treatment response. The need for accurate dosage and treatment duration in reproductive contexts is underscored by apprehensions over ovarian suppression or steroidogenic inhibition at supraphysiological levels, as shown by animal research.

#### 3.1.2. Natural Conception and Ovulation Outcomes

Data from the included randomised clinical studies indicate that GLP-1RAs significantly enhance the natural reproductive outcomes of overweight or obese women with PCOS. This is mainly due to the restoration of ovulation, the normalisation of hormonal profiles, and the correction of underlying metabolic abnormalities. The forthcoming randomised study by Liu et al. offers the most compelling clinical evidence among existing trials [29]. Participants demonstrated a significantly increased natural pregnancy rate (43.6% vs. 18.7%) in comparison to women undergoing metformin monotherapy, throughout the follow-up period after 12 weeks of treatment with exenatide (10 µg BID) followed by 12 weeks of metformin. Notably, improved menstrual regularity, decreased insulin resistance, and reduced testosterone levels coincided with better spontaneous conception, highlighting the connection between ovulatory recovery and metabolic optimisation.

Similarly, Salamun et al.’s randomised experiment over 12 months revealed that the combination of liraglutide and metformin improved both IVF and spontaneous pregnancy rates [28]. The persistent advantage after discontinuing GLP-1RA medication indicates that early metabolic reprogramming may trigger a series of durable improvements in ovarian function. The coordinated normalisation of these pathways likely promotes spontaneous ovulation, even in women previously deemed poor responders, as chronic anovulation in PCOS arises from a complex interplay of hyperinsulinemia, hyperandrogenism, disrupted granulosa-cell signalling, and impaired follicular maturation. Nylander et al. validate these findings, showing that liraglutide substantially increased the menstrual bleeding ratio and decreased free testosterone levels, both essential indicators of restored ovulatory function [30]. The reduction in ovarian volume seen in patients treated with liraglutide indicates a morphological shift towards a more physiological ovarian environment, consistent with improved follicular dynamics. Collectively, our data reveal a distinct pattern across trials: GLP-1RA therapy enhances the ovarian microenvironment essential for follicular selection and maturation, along with the endocrine circumstances that facilitate ovulation.

The noted clinical improvements correspond with recent discoveries from experimental models at a molecular level. GLP-1 and GLP-1 receptor agonists affect the expression of steroidogenic enzymes and modulate essential granulosa cell survival pathways, such as *FOXO1* phosphorylation and PI3K/AKT activation [25]. GLP-1RA signaling may facilitate the progression of follicles from preantral phases to dominant selection by suppressing apoptosis and diminishing oxidative and metabolic stress inside the follicular compartment. Furthermore, via reducing insulin-mediated CYP17A1 activation in theca cells, overall improvements in insulin sensitivity directly alleviate ovarian androgen excess. The ensuing decline in androgen levels augments aromatase activity and restores FSH-mediated granulosa cell function, thus elevating oestradiol synthesis and normalising the menstrual cycle [33]. Although the results are encouraging, it is crucial to acknowledge that the current clinical evidence is confined to women with PCOS and increased BMI. There is little data about normal-weight women, non-PCOS anovulation, hypothalamic dysfunction, or decreased ovarian reserve. The ideal time, dose, and duration of GLP-1RA treatment to improve natural fertility remain ambiguous. However, current evidence strongly suggests that GLP-1 receptor agonists may be an effective treatment for improving natural conception rates and restoring spontaneous ovulation in women with metabolic disorders, especially those with PCOS.

#### 3.1.3. Metabolic and Hormonal Outcomes

In all included clinical studies, GLP-1RAs consistently and substantially enhanced the metabolic and endocrine aspects contributing to reproductive issues in women with obesity and PCOS. These metabolic alterations facilitated ovulation, normal menstrual cycles, natural conception, and enhanced the efficacy of IVF. GLP-1RA medication resulted in substantial improvements in metabolic function. Liraglutide and exenatide markedly reduced body weight, total fat mass, and central adiposity. Nonetheless, only exenatide significantly reduced the proportion of visceral fat. Insulin sensitivity improved across all trials, shown by reduced fasting insulin levels, HOMA-IR, inflammatory markers, and lipotoxicity indices. The metabolic alterations focus on the principal variables that hinder conception in women with PCOS. Chronic hyperinsulinemia diminishes the production of hepatic SHBG and increases androgen levels via activating CYP17A1 in theca cells through insulin stimulation. GLP-1 receptor agonists efficiently inhibit the processes that elevate androgens by reducing blood insulin levels.

Endocrine enhancements strengthen this mechanism. Nylander et al. demonstrated clinically significant decreases in free testosterone and substantial increases in SHBG after 26 weeks of liraglutide therapy [30]. The reduction in bioavailable androgens is crucial for reinstating granulosa-cell differentiation, aromatase-mediated oestradiol production, and FSH sensitivity. Elevated testosterone levels alter FSHR expression, induce granulosa cell apoptosis, and inhibit follicular development, indicative of PCOS. Decreasing androgen levels enhances follicular growth and functionality. Subjects administered liraglutide exhibited reductions in ovarian volume, indicating a structural normalisation consistent with the restoration of ovulatory physiology.

Improvements in menstrual cyclicity were consistently seen throughout the studies. Liraglutide increased the bleeding ratio, but exenatide significantly raised the frequency of menstruation, both suggesting greater synchronisation of the HPO axis. In vitro and animal studies indicate that GLP-1 may modify the survival pathways of granulosa cells (*FOXO1*, PI3K/AKT), the expression of steroidogenic enzymes (StAR, P450scc, 3β-HSD), and the organism’s reaction to oxidative stress. These alterations presumably indicate that GLP-1 may rejuvenate metabolism across the body and perhaps have direct effects on the ovaries. The experiments demonstrated enhancements in hormones for reasons beyond weight reduction. Pregnancy outcomes enhanced proportionally to weight reduction in Salamun et al., indicating possible supplementary endocrine or intra-ovarian advantages. This aligns with previous mechanistic findings indicating that GLP-1R is present in granulosa cells and modulates cAMP-PKA signalling, which is crucial for follicular maturation induced by FSH. Consequently, enhanced mitochondrial activity, reduced oxidative stress, and increased granulosa cell proliferation may promote more effective follicular growth in vivo, as shown by experimental models.

### 3.2. Animal Study Findings

This paper provides valuable insights into the direct effects of GLP-1RA treatment on the ovaries, complementing the metabolic and clinical findings from human trials with in vivo animal investigations. The data indicates a diverse, dose-dependent, and context-sensitive range of ovarian responses, from possibly beneficial regulatory effects to significant histopathological changes, despite the limited number of current investigations. The research by Saber et al. offers the most comprehensive evaluation of uterine and ovarian morphology in female rats after liraglutide treatment [32]. Prolonged exposure resulted in a consistent pattern of ovarian disruption, characterised by granulosa-cell death, increased follicular atresia, vacuolar degeneration, and disorganisation of follicular architecture. Significant alterations occurred in the endocrine system, alongside these structural issues. Rats administered liraglutide had reduced levels of FSH, LH, oestradiol, and progesterone, while displaying elevated testosterone levels. This indicates that their hormones shifted to a more androgenic character and their ovaries ceased steroid production. The alterations were associated with indicators of heightened oxidative stress, including elevated malondialdehyde (MDA) levels and diminished antioxidant defences such as glutathione (GSH), superoxide dismutase (SOD), and catalase (CAT). This indicates that exposure to GLP-1RA may intensify oxidative damage in the ovarian milieu.

Despite the adverse effects, the research indicated that some might be ameliorated after the cessation of medication. This was shown by the improvement of several hormonal disorders and histological lesions throughout the recovery period. This study underscores the dynamic characteristics of GLP-1RA effects and the importance of exposure length when applied to human physiology. The findings from the research conducted by Sun et al. in a PCOS mouse model markedly contrasted with those of Saber et al. [18,32]. Under certain doses and metabolic settings, GLP-1RA exposure enhanced granulosa cell proliferation and reduced apoptosis by facilitating the phosphorylation and nuclear exclusion of *FOXO1*, a critical pro-apoptotic transcription factor. GLP-1 signalling may protect ovaries affected by metabolic imbalance. This is shown by the activation of supplementary signalling pathways linked to anti-inflammatory and antioxidant responses. The data indicate that the effects of GLP-1RA may differ markedly between ovaries exhibiting insulin resistance or PCOS and healthy ovarian tissue, suggesting that therapeutic advantages may be limited to certain metabolic conditions.

### 3.3. In Vitro Mechanistic Findings

This review’s in vitro findings provide critical mechanistic insights into the molecular effects of GLP-1 and its receptor agonists on granulosa cell function. These findings are significant as they elucidate whether the activation of GLP-1R has direct effects on the ovarian follicle and facilitate the investigation of signalling pathways independent of systemic metabolic influences. All in vitro findings indicate that GLP-1 signalling influences several intracellular mechanisms that regulate the development, apoptosis, steroidogenesis, and responsiveness of granulosa cells to gonadotropins. Sun et al. performed a comprehensive mechanistic investigation, demonstrating that the activation of GLP-1R in granulosa cells results in the phosphorylation of the transcription factor *FOXO1*, leading to its eventual exclusion from the nucleus. Translocating *FOXO1* from the nucleus diminishes the expression of pro-apoptotic genes and enhances cell survival, since it serves as a crucial regulator of apoptosis, cell-cycle arrest, and metabolic stress responses in granulosa cells. In a biological setting similar to PCOS, GLP-1RA administration promoted granulosa cell proliferation and provided resistance to apoptosis via this route. These results indicate that GLP-1 receptor agonists may improve follicular health in insulin-resistant or hyperandrogenic ovaries by restoring proliferative capability and diminishing intrinsic apoptotic stress.

Nishiyama et al.’s study on the involvement of incretins, such as GLP-1 and GIP, in steroidogenesis control reveals more molecular complexities [31]. Their research shown that GLP-1 inhibits granulosa cells from producing progesterone in response to FSH stimulation by reducing the activity of key steroidogenic enzymes such as 3β-HSD, P450scc, and StAR. The observed effects, associated with reduced intracellular cAMP levels, indicated a potential inhibitory interaction between GLP-1R signalling and the conventional FSH–cAMP–PKA pathway, which facilitates luteinisation and progesterone synthesis. Both GLP-1 and GIP reduced progesterone levels. On the other hand, they had divergent effects on BMP-Smad signalling. GIP, unlike GLP-1, enhanced the activation of Smad1/5/8 induced by BMP-6. This indicates that BMP receptor signalling may be altered by various ligands. This selective interaction may elucidate the divergent steroidogenic effects of GLP-1 and GIP, since BMP-Smad pathways are acknowledged to inhibit progesterone production. The intricacies highlight that intracellular context, ligand specificity, and receptor expression all influence incretin signalling in granulosa cells.

In vitro results indicate that GLP-1 signalling regulates the expression of BMP Type-I receptors, namely ALK-3 and ALK-6. These receptors subsequently alter the response of granulosa cells to local ovarian growth stimuli. Granulosa cells treated to GLP-1 or GIP exhibit increased sensitivity of their receptors to BMP-mediated suppression of steroidogenesis. The alterations, along with a reduction in the expression of inhibitory Smad6, indicate that incretin signalling is integral to a complex regulatory framework that modifies the equilibrium between BMP-mediated inhibition and gonadotropin-induced progesterone synthesis. Besides steroidogenesis and apoptosis, GLP-1 signalling also influences oxidative stress responses. Data from comparable models suggest that GLP-1/GLP-1RA administration affects mitochondrial activity, the production of reactive oxygen species, and the expression of antioxidant enzymes; this has not been definitively shown by direct in vitro research. These molecular pathways need more examination, since oxidative equilibrium is essential for granulosa cell functionality and oocyte competence.

### 3.4. Integrated Synthesis of Clinical, Animal, and In Vitro Findings

This review of clinical, animal, and in vitro research presents a complex and context-dependent overview of the impact of GLP-1RAs on the female reproductive system. In human clinical studies, GLP-1RA medication consistently improved metabolic status, reduced androgen excess, restored menstrual cyclicity, increased ovulation rates, and boosted both spontaneous and assisted conception results. The benefits were sometimes disproportionate to the extent of weight reduction, indicating supplementary reproductive processes beyond just metabolic adjustment. The results illustrate the therapeutic advantages of GLP-1 receptor agonists for women with PCOS and metabolic dysfunction, whereby anovulation and diminished fertility are mostly associated with insulin resistance and hyperandrogenism.

The molecular and cellular insights derived from in vitro studies provide the mechanistic foundation for these clinical progressions. Investigations involving granulosa cells reveal that GLP-1 signalling affects essential intracellular pathways governing apoptosis, proliferation, and steroidogenesis. Activation of GLP-1R prevents the apoptosis of granulosa cells by phosphorylating *FOXO1*, therefore sequestering it in the cytoplasm and excluding it from the nucleus. It also interacts with the cAMP-PKA and BMP-Smad pathways to maintain equilibrium between FSH-induced steroidogenesis and local intra-ovarian signalling. The incretin-mediated modulation of enzymes such as StAR, P450scc, and 3β-HSD clarifies clinical endocrine improvements, including the normalisation of SHBG, decrease in free testosterone, and augmentation of ovulatory dynamics.

In vivo animal studies elucidate these molecular results and illustrate the dualistic nature of GLP-1RA activity. GLP-1 signalling enhanced follicular health in models that were metabolically impaired or analogous to PCOS, aligning with the observed benefits in individuals with PCOS. The impact of GLP-1RA is significantly influenced by metabolic condition, length of therapy, and physiological setting. Studies on healthy rats given supra-physiological dosages of liraglutide demonstrated ovarian disturbance, indicated by increased follicular atresia, oxidative damage, hormonal suppression, and structural disorganisation. This disparity suggests that GLP-1 receptor agonists may be advantageous for insulin-resistant ovaries but perhaps detrimental to normal ovarian tissue under certain circumstances. This review presents a complex and context-dependent overview of the impact of GLP-1RAs on the female reproductive system, as shown by clinical, animal, and in vitro investigations. In human clinical studies, GLP-1RA medication consistently improved metabolic status, reduced androgen excess, restored menstrual cyclicity, increased ovulation rates, and boosted both spontaneous and assisted conception results. The benefits were sometimes disproportionate to the extent of weight reduction, indicating supplementary reproductive processes beyond just metabolic adjustment. The results illustrate the therapeutic advantages of GLP-1 receptor agonists for women with PCOS and metabolic dysfunction, whereby anovulation and diminished fertility are mostly associated with insulin resistance and hyperandrogenism.

The molecular and cellular insights derived from in vitro studies provide the mechanistic foundation for these clinical improvements. Investigations involving granulosa cells reveal that GLP-1 signalling affects essential intracellular pathways governing apoptosis, proliferation, and steroidogenesis. Activation of GLP-1R prevents the apoptosis of granulosa cells by phosphorylating *FOXO1*, therefore sequestering it from the nucleus. It also interacts with the cAMP-PKA and BMP-Smad pathways to maintain the equilibrium between FSH-mediated steroidogenesis and local intra-ovarian signalling. The modulation of enzymes such as StAR, P450scc, and 3β-HSD by incretins clarifies clinical endocrine improvements, including the normalisation of SHBG, a decrease in free testosterone, and promotion of ovulatory processes.

In vivo animal studies elucidate these molecular results and illustrate the dualistic nature of GLP-1RA activity. GLP-1 signalling enhanced follicular health in animals exhibiting metabolic compromise or characteristics akin to PCOS, aligning with the observed benefits in individuals with PCOS. The effects of GLP-1RA are significantly influenced by metabolic state, length of therapy, and physiological setting. Investigations on healthy rats given supra-physiological dosages of liraglutide indicated ovarian disturbance, characterised by increased follicular atresia, oxidative damage, hormonal suppression, and structural disorganisation. This disparity indicates that GLP-1 receptor agonists may assist ovaries unresponsive to insulin; they may adversely affect normal ovarian tissue in some instances. The main Findings of our research are presented on Table 3.

## 4. Discussion

When reading this systematic review, you should remember how GLP-1 receptor agonists have changed the way reproductive medicine works. Early integrative frameworks identified metabolic correction as the primary mediator of reproductive advantage, especially in obese women with PCOS. The current synthesis demonstrates that GLP-1 receptor agonism affects ovarian biology on various levels, including FOXO1-dependent granulosa cell survival, modulation of FSH-cAMP-BMP signalling, and context-specific impacts on steroidogenesis, despite the ongoing relevance of this paradigm. Our review enhances previous integrative studies by synthesising animal models, mechanistic cellular data, and IVF outcomes to highlight the therapeutic potential and biological limitations of GLP-1 receptor agonists. We need a more nuanced understanding of ovarian responsiveness, dose dependency, and reproductive safety that takes into account the specific situation and not just metabolic factors.

The growing investigation of GLP-1RAs in human reproduction uncovers a complicated and sometimes contradictory molecular background. Mechanistic research using in vitro systems and animal models, particularly those by Sun, Nishiyama, Xiong, Saber, and Yin demonstrate that GLP-1RA signalling has significantly context-dependent effects on reproductive organs [18,31,32,34,35]. Under metabolic stress situations such as PCOS, GLP-1R activation may promote granulosa cell proliferation, elevate *FOXO1* phosphorylation, and diminish apoptosis, as seen by these studies. Nevertheless, prolonged exposure to metabolically normal ovaries may inhibit progesterone synthesis, alter BMP-Smad signalling, elevate oxidative stress, and induce structural damage to follicles. The data suggest that GLP-1 receptor agonists may have both advantageous and adverse effects, depending on the specific ovarian or extra-ovarian compartment, length of drug exposure, and the individual’s metabolic profile [36].

A far more consistent and beneficial trend is seen in obese women with PCOS when examining the available clinical data from randomised trials, observational cohorts, and meta-analyses. The therapeutic effectiveness of GLP-1 receptor agonists in this population is robustly corroborated by the consistent results of Zhou [37]. GLP-1RA medication consistently boosts monthly regularity, elevates ovulation rates, reduces testosterone levels, diminishes central adiposity, improves insulin sensitivity, and increases spontaneous conception rates across diverse trials with varying demographics and methodology [38]. The randomised findings from Salamun and Liu demonstrate that GLP-1 receptor agonists may significantly enhance spontaneous conception rates. Salamun also indicates a significant increase in the incidence of pregnancies occurring subsequent to each embryo transfer during IVF [37]. This suggests that GLP-1RA has benefits beyond only improving metabolism and may potentially affect the physiology of the ovaries or endometrium. Nylander’s findings corroborate this reproductive advantage by demonstrating a reduction in ovarian volume, an increase in the bleeding ratio, and elevated levels of SHBG and free testosterone [30]. These studies also link the restoration of ovarian function to improved metabolism.

### 4.1. Molecular and Cellular Mechanisms of GLP-1RA Action: Integrating In Vitro, Animal, and Human Evidence

Examining the molecular data from Sun, Nishiyama, Xiong, Saber, and Yin reveals that GLP-1 receptor agonists have both analogous and divergent effects on ovarian and reproductive organs [18,31,32,34,35]. Sun et al. assert that GLP-1 amplifies anti-apoptotic signalling and reinstates metabolic resilience in insulin-resistant follicles via the phosphorylation-mediated silencing of *FOXO1*, thereby fostering granulosa cell proliferation in the ovaries of PCOS mice [18]. The findings of Saber et al. demonstrated considerable granulosa-cell death, follicular atresia, oxidative stress, and total inhibition of gonadotropin-dependent steroidogenesis in metabolically normal rats after prolonged liraglutide administration, which sharply contrasts with this profile [32]. Saber et al. propose that liraglutide may function as an ovarian toxin in physiological contexts, whereas Sun et al. characterise GLP-1RA signalling as a protective mechanism for metabolically dysregulated follicles [32].

Comparing the GLP-1–*FOXO1* model by Sun et al. with the BMP–Smad modulation by Nishiyama et al. elucidates their distinctions more clearly [18,31]. Nishiyama’s study demonstrates that incretin signalling, particularly GIP and, to a lesser extent, GLP-1, suppresses StAR, P450scc, and 3β-HSD, hence diminishing progesterone production while concurrently enhancing Smad1/5/8 phosphorylation in response to BMP-6 [31]. These results clearly contradict the findings of Sun et al., which revealed that GLP-1 promotes healthy follicular survival in PCOS via *FOXO1*-dependent anti-apoptotic mechanisms. This research outlines a molecular route via which GLP-1RA administration reduces FSH responsiveness and luteal differentiation. The comparison reveals that the metabolic environment of PCOS vs. normal ovary significantly affects the progression of GLP-1 signalling concerning granulosa cell fate and steroidogenesis.

Xiong et al. revealed that liraglutide and semaglutide modify the gut microbiota in mice with DHEA-induced PCOS [34]. They do this by increasing butyrate-producing taxa, restoring microbial diversity, and improving androgenic and metabolic indices, thereby confounding the process. This microbiota-mediated mechanism, in conjunction with *FOXO1* signalling and BMP inhibition, introduces a third mechanistic dimension by suggesting that GLP-1 receptor agonists influence ovarian function via metabolic communication between the gut and the ovaries [39]. The study by Sun, Nishiyama, and Saber does not include this method [18,31,32]. Xiong’s research indicates that the advantages of GLP-1RA in PCOS may derive from systemic metabolic reconfiguration driven by microbial metabolites, especially short-chain fatty acids that influence insulin sensitivity and inflammatory response, alongside direct ovarian impacts [34]. Yin et al. enhanced the mechanistic framework by investigating male reproductive tissues and finding that neither semaglutide nor liraglutide adversely affected testicular morphology, motility, or sperm count in elderly or diabetic mice [35]. Their findings provide a relevant comparison to the female-focused data reported by Saber et al., albeit concentrating on male reproductive physiology [32]. Gonadal toxicity is neither ubiquitous or unavoidable. It may be an ovarian-specific occurrence shaped by follicular physiology and sex-specific steroidogenic processes. In males, exposure to GLP-1 receptor agonists seems to be neutral and non-harmful, even under metabolic stress [35].

Compiling all mechanistic information reveals a consistent pattern: GLP-1 receptor agonists may disrupt normal ovarian function when administered to healthy animals over an extended duration [40]. Nevertheless, they often reinstate cellular function when metabolism is impaired in PCOS models. The metabolic improvements provided by microbiota, as shown by Xiong et al., together with the *FOXO1*-mediated anti-apoptotic effects observed by Sun et al., jointly confirm therapeutic benefits in PCOS biology [34]. The oxidative stress-induced follicular degeneration identified by Saber et al. and the progesterone suppression shown by Nishiyama et al. highlight concerns about the potential impact of GLP-1 receptor agonists on ovarian function in euglycemic, non-PCOS mice [31,32].

### 4.2. Clinical Evidence: Integrating IVF, Natural Conception, and Endocrine-Metabolic Outcomes Across Human Studies

In contrast to the contradicting molecular and animal results, clinical research investigating the reproductive effects of GLP-1 receptor agonists in women with PCOS reveals a notably consistent pattern of benefit [41]. A consolidated clinical signal from clinical trials, meta-analyses, and narrative reviews indicates that GLP-1 receptor agonists routinely enhance metabolic dysfunction, menstrual regularity, ovulation, and, in several instances, spontaneous conception rates in overweight or obese individuals with polycystic ovary syndrome [42]. Despite this convergence, studies demonstrate considerable diversity in the strength, nature, and reproductive implications of these benefits, requiring a nuanced synthesis. The randomised trials by Salamun et al. and Liu et al. demonstrate significantly increased natural pregnancy rates in obese women with PCOS after short-term GLP-1RA medication, providing the most persuasive clinical evidence for reproductive benefit. Salamun et al. discovered that the combination of metformin and low-dose liraglutide resulted in an impressive 85.7% pregnancy rate per embryo transfer [28,29]. This was almost thrice more than the rate seen with metformin alone. This is consistent with, but also contrasts with, the results of Liu et al., who indicated that exenatide pretreatment more than quadrupled the incidence of natural conception in comparison to metformin monotherapy [29]. A comparison of these two studies indicates a significant pattern: weight reduction was comparable across groups in Salamun et al. and only partly informative in Liu et al., indicating that both liraglutide and exenatide provide reproductive improvements that cannot be solely attributed to weight loss.

Meta-analytic evidence from Zhou et al. and De Hollanda Morais et al. corroborates these results, indicating that GLP-1 receptor agonists substantially decrease BMI and central adiposity while improving monthly regularity, ovulation likelihood, SHBG levels, and free testosterone levels [37,43]. Zhou et al. could not detect a notable increase in IVF pregnancy rates within aggregated datasets [37]. They specifically noted that GLP-1RA medication improves natural pregnancy rates (RR 1.72), which is consistent with the individual results of Liu and Salamun [28,29]. This disparity underscores a critical comparative aspect: although Salamun et al. identified a notable IVF advantage, this effect may be contingent upon particular population characteristics (severe obesity, poor responders) or transient metabolic optimisation prior to IVF, which may not be consistently observed in larger, more heterogeneous samples utilised in meta-analyses [28]. Nylander et al.’s recent findings support the notion that GLP-1 receptor agonists alter ovarian physiology beyond just facilitating weight loss [30]. Nylander documented benefits in bleeding ratio, ovarian volume, SHBG, and free testosterone, while metabolic advantages were minimal compared to previous GLP-1RA studies [30]. In contrast to Salamun and Liu, Nylander’s research highlights the possibility of a direct relationship between GLP-1 signalling and ovarian endocrine function, indicating that indicators of ovarian function (bleeding pattern, androgenic profile, and ovarian size) may improve even in the absence of significant weight loss [28,29,30].

Narrative and clinical reviews, such as those by Hoteit et al., Goldberg & Boots, and Varughese et al., provide more comprehensive interpretative frameworks [22,38,44]. These studies together demonstrate that GLP-1RAs address many pathophysiological mechanisms essential to PCOS, including insulin resistance, chronic inflammation, hypothalamic ovarian dysregulation, and metabolic dysfunction. In conjunction with Sun et al.’s mechanistic evidence on *FOXO1*-mediated survival and Xiong et al.’s microbiome-related benefits, Hoteit et al. contend that GLP-1 receptor agonists may have a “direct role” in ovarian morphology and the restoration of ovulation [18,22,34]. A synergy between direct ovarian signalling and metabolic correction is suggested by the discovery that, when these reviews are compared with individual clinical trials, doctors consistently report improvements in reproductive outcomes, regardless of the diversity in weight reduction. Varughese et al. and Goldberg & Boots emphasise the rising incidence of “Ozempic pregnancies,” demonstrating that exposure to GLP-1RA before conception often results in spontaneous ovulation and unwanted births, despite a lack of official clinical evidence [38,44]. These signs, however anecdotal, support the concept that GLP-1RA medication may restore ovulatory function in women with PCOS who were previously anovulatory, consistent with the systematic findings provided by Salamun, Liu, and Zhou [28,29,37].

Evidence suggests that body mass index independently influences assisted reproduction results in women with PCOS, highlighting clinical heterogeneity. Su et al. found that pregnancy outcomes after blastocyst transfer varied significantly across BMI categories in PCOS patients receiving ultralong GnRH-a protocols, despite uniform diagnostic criteria and standardised treatment methods [45]. This discovery is especially important when assessing IVF results after GLP-1 receptor agonist pretreatment, since variations in reproductive success may be linked to improvements in metabolic condition rather than just to the diagnosis of PCOS.

### 4.3. Metabolic and Hormonal Mechanisms Underlying the Clinical Benefits of GLP-1RAs

Clinical research investigating the reproductive effects of GLP-1 receptor agonists in women with PCOS reveals a notably consistent pattern of benefit, in contrast to the more ambiguous mechanistic and animal evidence [46]. A consolidated clinical signal from clinical trials, meta-analyses, and narrative reviews indicates that GLP-1 receptor agonists consistently enhance metabolic dysfunction, menstrual regularity, ovulation, and, in many instances, natural conception rates in overweight or obese individuals with PCOS. Despite this convergence, research reveals considerable diversity in the nature, quantity, and reproductive implications of these advantages, requiring a nuanced synthesis [22,47].

The most persuasive clinical evidence for reproductive advantage comes from the randomised trials by Salamun et al. and Liu et al., both of which reveal significantly increased natural pregnancy rates in obese women with PCOS after short-term GLP-1RA medication [28,29]. Salamun et al. reported that the combination of metformin and low-dose liraglutide achieved an impressive 85.7% pregnancy rate per embryo transfer [28]. This risk was almost thrice greater than the rate associated with metformin alone. Liu et al. discovered that exenatide pretreatment more than quadrupled the natural conception rate in comparison to metformin monotherapy [29]. This resembles our findings; however, it is not identical. A significant pattern arises from the comparison of these two trials: weight decrease was comparable across the groups in Salamun et al. and only partly elucidated in Liu et al., indicating that both liraglutide and exenatide provide reproductive improvements that cannot be solely attributed to weight loss [28,29].

Meta-analytic evidence from Zhou et al. and De Hollanda Morais et al. corroborates these results, indicating that GLP-1 receptor agonists improve monthly regularity, increase the probability of ovulation, elevate SHBG levels, and decrease free testosterone, while markedly lowering BMI and central obesity [37,43]. Zhou et al. expressly indicated that GLP-1RA medication enhances natural pregnancy rates (RR 1.72), which is consistent with the individual results of Liu and Salamun [28,29,37]. However, they did not detect a significant increase in IVF pregnancy rates among aggregated datasets. This inconsistency underscores a critical comparative aspect: although Salamun et al. recognised a substantial IVF advantage, this effect may depend on particular population characteristics (severe obesity, poor responders) or short-term metabolic optimisation prior to IVF, which may not be consistently observable in larger, more heterogeneous samples employed in meta-analyses [28]. Nylander et al.’s recent results support the notion that GLP-1 receptor agonists alter ovarian function beyond just facilitating weight loss [30]. Nylander documented gains in bleeding ratio, ovarian volume, SHBG, and free testosterone, with only slight metabolic advancements compared to previous GLP-1RA studies [30]. In contrast to Salamun and Liu, Nylander’s research highlights the possibility of a direct relationship between GLP-1 signalling and ovarian endocrine function, indicating that markers of ovarian function (including bleeding patterns, androgenic profiles, and ovarian size) may improve even without significant weight loss [28,29,30].

Narrative and clinical reviews, such as those by Hoteit et al., Goldberg & Boots, and Varughese et al., provide more extensive interpretative frameworks [22,38,44]. The reviews together suggest that GLP-1RAs target many pathophysiology components of PCOS, including metabolic dysfunction, insulin resistance, chronic inflammation, and hypothalamic-ovarian dysregulation. Hoteit et al. contend that GLP-1 receptor agonists may have a “direct role” in ovarian morphology and the restoration of ovulation, supporting the mechanistic results of Sun et al. regarding *FOXO1*-mediated survival and the microbiome-related advantages identified by Xiong et al. [18,22,34]. The observation that clinicians consistently report improvements in reproductive outcomes when these reviews are compared with individual clinical trials, regardless of variations in weight loss, suggests a synergistic relationship between direct ovarian signalling and metabolic correction. Despite the absence of official clinical data, Varughese et al. and Goldberg & Boots discuss the increasing incidence of “Ozempic pregnancies” [38,44]. Exposure to GLP-1RA before conception often results in spontaneous ovulation and unintended pregnancies. These signs, however anecdotal, support the systematic findings shown by Salamun, Liu, and Zhou, suggesting that GLP-1RA medication may restore ovulatory function in women with PCOS who were previously anovulatory [28,29,37].

Non-pharmacological metabolic interventions have demonstrated a disconnection between metabolic enhancement and reproductive results. A recent systematic review and meta-analysis by Turetta et al. found that ketogenic dietary interventions significantly improve weight, insulin sensitivity, and endocrine parameters in women with PCOS [48]. However, reproductive outcomes are still variable and depend on the phenotype. These results support findings with GLP-1 receptor agonists, indicating that metabolic normalisation alone does not consistently lead to improved ovarian response or fertility. They also show how important it is to have personalised treatment plans.

### 4.4. Safety Considerations, Endometrial Receptivity, and Knowledge Gaps

It is essential to recognise the discrepancies and partial inconsistencies between animal and human evidence regarding the reproductive effects of GLP-1 receptor agonists when examining the current information. Numerous animal investigations have shown that GLP-1RA administration may induce ovarian suppression, granulosa cell death, and increased oxidative stress. Clinical investigations including women with obesity or PCOS have consistently shown metabolic and reproductive advantages.

The observed variances are likely attributable to fundamental variations in ovarian physiology between species, their baseline metabolic conditions, the administration methods of the dosages, and the duration of exposure. Animal studies sometimes use prolonged treatment periods or supraphysiological dosages, which may not adequately represent therapeutic applications in humans. Furthermore, human research often concentrates on insulin-resistant or obese populations, where GLP-1 signalling may have protective rather than inhibitory effects on ovarian function, whereas experimental models frequently use metabolically healthy animals. Therefore, the straight application of animal safety findings to human reproductive medicine requires prudence. Animal research raises significant mechanistic and toxicological issues. Ιt should be seen as hypothesis-generating rather than conclusive proof of human reproductive danger. Comprehending dose-dependent effects, treatment timing, and reproductive safety across many metabolic profiles necessitates well-conducted, long-term human investigations.

The safety profile of GLP-1RAs in reproductive situations is little clarified, despite their notable therapeutic benefits in obese women with PCOS. A notable pattern reveals that efficacy and risk are highly context-dependent, influenced by metabolic phenotype, timing of drug exposure, dosage, and the physiological state of the ovary and endometrium, when juxtaposing reproductive safety data from human trials with animal studies and mechanistic in vitro research [43,49]. Saber et al.’s preclinical research indicated that extended exposure to liraglutide causes considerable ovarian damage in metabolically normal rats, marked by granulosa cell apoptosis, follicular atresia, vacuolar degeneration, and significant oxidative stress, thereby raising serious safety concerns [32]. These data highlight that ovarian response is affected by metabolic state, markedly differing from the beneficial granulosa-cell survival effects reported by Sun et al. in PCOS mouse models [18]. In healthy ovaries, GLP-1 receptor agonists may suppress gonadotropin-dependent steroidogenesis [50]. However, in metabolically compromised PCOS tissue, as demonstrated by Sun, the same pathway enhances functionality, as indicated by Saber’s findings of diminished FSH, LH, oestradiol, and progesterone levels, coupled with increased testosterone [18,32]. This disparity indicates a significant knowledge gap: The ovarian safety of GLP-1RA in normal-weight, non-PCOS women of reproductive age in humans is essentially uncharacterized.

Nishiyama et al.’s mechanistic study raises more questions about the functioning of the luteal phase [31]. Their in vitro study demonstrates that incretins inhibit progesterone production by augmenting the BMP Smad1/5/8 signalling pathway and down-regulating StAR, P450scc, and 3β-HSD. In some cases, these molecular abnormalities may hinder luteal competency or implantation. The mechanistic data clarify a biologically plausible pathway through which peri-ovulatory or pre-luteal GLP-1RA exposure may affect steroidogenesis, a gap frequently highlighted by endometrial researchers, despite the lack of human clinical trials showing progesterone deficiency during GLP-1RA administration. Sola-Leyva et al. emphasise a critical aspect, noting that the impact of GLP-1 receptor agonists on endometrial receptivity and implantation is mostly unclear [46]. The endometrium’s reaction has not been examined in controlled human studies, in contrast to the metabolic effects on the ovaries. Sola-Leyva contends that modified decidualisation, stromal cell signalling, immune cell recruitment, and early implantation biology are areas of considerable molecular sensitivity where the impacts of GLP-1RA may be either neutral or adverse [46]. No research has differentiated the effects of the endometrium from those of the ovaries and metabolism. Nevertheless, the increased IVF pregnancy rates seen in the Salamun et al. study may indicate improved endometrial receptivity after metabolic augmentation. Thus, it is unclear whether GLP-1RAs have a direct effect on the human endometrium [28].

Comprehensive safety studies in clinical reviews elucidate the ambiguity further. Varughese et al. and Goldberg & Boots both indicate that an increasing number of women of reproductive age are being exposed to GLP-1 receptor agonists in real-world scenarios, sometimes referred to as “Ozempic pregnancies” [38,44]. Such exposures often result in unplanned births soon after the initiation of therapy. No teratogenic effects in humans are documented. Manufacturers advise ceasing medication at least one to two months before conception, owing to the lack of controlled research on prenatal exposure to evaluate safety during early embryogenesis [51]. Varughese et al. highlight a significant translational gap, since the majority of evidence about foetal safety is derived from animal toxicity studies conducted at levels too high for humans [38]. Research by Nylander and Zhou demonstrates that overweight women with PCOS do not exhibit cycle suppression or luteal irregularities, offering confidence to reproductive endocrinologists that medication improves menstrual regularity and ovarian function [30,37]. Yin et al.’s study on male reproduction indicates that GLP-1 receptor agonists do not adversely affect gonadal function in either gender [35]. Even at high dosages, they do not adversely affect sperm parameters. However, these results do not apply to women experiencing early embryo implantation or to healthy, thin women.

The independent and combined effects of polycystic ovarian syndrome and obesity on reproductive dysfunction provide a notable source of variability that requires further investigation. Recent clinical data suggest that obesity and PCOS are separate pathological disorders that uniquely affect metabolic, endocrine, and ovarian characteristics, rather than forming a solitary, cohesive phenotype. Vale-Fernandes et al. contend that both PCOS and body weight adversely influence fertility treatment results, while their interaction further diminishes reproductive success [52]. These results highlight the need for a customised paradigm in evaluating the reproductive effects of GLP-1 receptor agonists, suggesting that metabolic state and inherent ovarian disease should be assessed independently when analysing reproductive outcomes.

The influence of GLP-1 receptor agonists on luteal function, endometrial receptivity, ovarian activity, and metabolism is a significant unsolved matter in reproductive medicine. Despite evidence that pretreatment with GLP-1RA enhances conception and implantation rates, particularly in women with PCOS, the underlying mechanisms for these advantages remain unclear. The reason remains ambiguous, whether due to direct impacts on the endometrium, indirect metabolic alterations, or enhanced oocyte and embryo competence. Mechanistic facts on luteal steroidogenesis need more substantial investigations. In vitro data suggests that the manipulation of the FSH-cAMP and BMP- Smad pathways may suppress progesterone production; this phenomenon has not been well investigated in people. This indicates that luteal adequacy may be affected by exposure to GLP-1 receptor agonists during the periovulatory or luteal phase.

Therefore, future studies should focus on the direct evaluation of endometrial receptivity, including timed endometrial biopsies with transcriptomic and proteomic analysis, assessment of decidualisation markers, immune cell composition, and signalling pathways related to implantation. Clinical investigations investigating luteal hormone dynamics, implantation rates, and early pregnancy outcomes after controlled GLP-1RA administration are crucial for evaluating safety, timing, and reproductive consequences. To safely and rationally include GLP-1 receptor agonists into reproductive therapy protocols, these deficiencies must be addressed.

The information combined reveals many substantial safety issues and unsolved enquiries about the use of GLP-1 receptor agonists in reproduction. The reproductive safety of GLP-1 receptor agonists in metabolically healthy, normal-weight women is mainly unexplored, since existing human clinical data mostly concentrate on women with obesity or polycystic ovary syndrome. Secondly, interspecies differences and non-physiological dose limit the translational relevance of preclinical animal studies that raise concerns about ovarian suppression, oxidative stress, and altered steroidogenesis due to prolonged or heightened exposure. The impact of GLP-1 receptor agonists on human endometrial receptivity, luteal function, and early implantation has not been well assessed. Ultimately, it is unclear when, for how long, and to what degree GLP-1RA medication should be used in relation to ovarian stimulation and conception. These limitations highlight the need for carefully structured, phenotype-specific clinical studies before approving the widespread use of GLP-1 receptor agonists in reproductive medicine.

### 4.5. Future Directions and Translational Perspectives

The substantial therapeutic promise of GLP-1 receptor agonists in reproductive medicine, especially for obese women with PCOS, is highlighted by the amalgamation of clinical, molecular, and animal data. However, the inconsistent results between preclinical and human studies highlight the need for focused translational research that explains molecular pathways pertinent to ovarian and endometrial physiology, identifies risk groups, and specifies therapy parameters.

It is essential to establish extensive, multicenter randomised studies to evaluate GLP-1 receptor agonists in women undergoing reproductive therapy. The current studies are constrained by modest sample sizes, brief treatment durations, and diverse reproductive outcomes. Research by Salamun, Liu, Nylander, and the meta-analysis by Zhou and De Hollanda Morais consistently demonstrate improvements in ovulation, menstrual regularity, natural conception rates, and, in some cases, IVF results [28,30,37,43]. To provide meaningful cross-study comparisons, next clinical research must use defined reproductive outcomes, including oocyte quality, ovulation rates, luteal adequacy, endometrial thickness and morphology, implantation rate, biochemical pregnancy, clinical pregnancy, and live birth rates. Furthermore, as shown by Salamun, procedures assessing short-term, preconception GLP-1RA exposure prior to IVF cycles require comprehensive validation, as they may function as an efficacious metabolic-priming technique to improve ovarian response and endometrial receptivity [28].

A secondary translational goal is the methodical investigation of endometrial effects, which Sola-Leyva et al. have consistently identified as a significant knowledge need [46]. Despite therapeutic studies routinely showing enhanced reproductive results, none have shown whether GLP-1RA signalling directly affects the endometrium. Molecular studies must assess decidualisation markers, endometrial stromal cell responses, immune cell recruitment, cytokine expression, and genes associated with implantation (LIF, integrins, cytokines, HOXA10, IGFBP1). We can ascertain whether GLP-1RAs exert neutral, positive, or detrimental effects on the implantation cascade only using biopsy-derived or organoid-based models. Nishiyama’s mechanistic signals revealed incretin-mediated suppression of progesterone production, a pathway with considerable ramifications for luteal function and endometrial receptivity, highlighting the significance of this discovery [31].

Another method to interpret the data is to elucidate the dose-response and metabolic-context dependence in animal and in vitro research. The detrimental oxidative stress and follicular degeneration identified by Saber et al. in healthy rats starkly contrast with the protective benefits conferred by *FOXO1* phosphorylation shown by Sun et al. in PCOS models [18,32]. Future research should examine the effects of insulin resistance, obesity, inflammation, and oxidative stress on ovarian responses to GLP-1 receptor agonists. Ovary-on-a-chip models, ex vivo follicular cultures, and human in vitro studies employing granulosa cells from both PCOS and non-PCOS patients could clarify whether the clinically observed ovarian benefits are dependent on metabolic disruption or if GLP-1RAs affect ovarian physiology independently of systemic metabolic conditions.

Subsequent research should examine the gut ovary axis as a mechanistic element contributing to the reproductive benefits associated with GLP-1 receptor agonists, in light of recent discoveries by Xiong et al. on gut microbiota modulation [34]. Assessments of short-chain fatty acids, metagenomic analysis, and investigations of faecal transplantation may clarify if modifications in microbiota have a causal role in linking GLP-1RA medication to enhanced ovulation, decreased testosterone levels, and rejuvenated follicular function. The microbiota results may indicate potential synergistic reproductive benefits of combining GLP-1 receptor agonists with dietary, probiotic, or metabolic-targeting treatments.

Varughese, Goldberg, and Sola-Leyva have emphasised that the long-term safety for the unborn and periconception is a critical area of translational significance that requires more investigation [38,44,46]. There is an urgent need for regulated pregnancy-exposure registries because to the rise in real exposure, often known as “Ozempic pregnancies.” These should record foetal growth, placental morphology, early embryonic developmental outcomes, neonatal metabolic health, and long-term developmental trajectories in children conceived during or soon after GLP-1RA exposure. The periconceptional window is very sensitive and requires comprehensive examination because of the endocrine and implantation-related biochemical pathways involved.

The development of next-generation GLP-1-based therapies introduces a promising new domain of study. Multireceptor incretin agonists (GLP-1/GIP dual agonists, GLP-1/GIP/glucagon triagonists) may provide greater metabolic improvements relative to liraglutide or semaglutide, thereby increasing reproductive results. Nonetheless, they may have unique ovarian effects and more complex pathways. Translational research must emphasise comparative assessments of ovarian steroidogenesis, follicular dynamics, and endometrial function in PCOS models, paving the way for future clinical trials.

These translational goals combined indicate a growing recognition that GLP-1 receptor agonists serve as systemic metabolic regulators with significant reproductive implications, beyond their function as weight-loss medicines. Robust mechanistic and clinical research is crucial to define the ideal function of these drugs in augmenting fertility, boosting IVF results, and ensuring safe conception, especially as their use among women of reproductive age increases fast. In the next decade, we will ascertain if GLP-1 receptor agonists are exclusively beneficial for certain metabolic abnormalities, such as PCOS, or whether they will be integrated as a standard approach to enhancing reproductive health.

## 5. Conclusions

This comprehensive review compiles all clinical, experimental, and mechanistic evidence on the impact of glucagon-like peptide-1 receptor agonists on reproduction. Data suggest that GLP-1RAs may provide significant reproductive benefits to women with metabolic dysfunction and polycystic ovary syndrome, including improved ovulation and menstrual regularity, as well as, in certain instances, better outcomes for both natural and assisted conception. These benefits seem to extend beyond simple weight reduction, perhaps affecting granulosa-cell survival, hormonal balance, and ovarian signalling pathways directly.

Clinically, our data indicate that GLP-1 receptor agonists may be a valuable supplementary therapy for some infertile individuals with obesity and PCOS, especially when metabolic abnormalities are implicated in reproductive failure. Nonetheless, restricted sample sizes, brief follow-up periods, and significant research variability continue to limit the available data.

The reproductive implications of GLP-1 receptor agonists in metabolically healthy, non-PCOS women are mostly unexamined, and preclinical evidence suggests possible safety issues in certain scenarios. In reproductive medicine, GLP-1 receptor agonists should be used judiciously, considering the patient’s phenotype, treatment scheduling, and duration of therapy.

Prior to recommending extensive clinical use, rigorously structured, longitudinal human investigations concentrating on ovarian function, endometrial receptivity, luteal adequacy, and early pregnancy outcomes are needed. In the absence of more proof, GLP-1 receptor agonists should be regarded as promising but experimental instruments for fertility management.

## Figures and Tables

**Table 1:** Quality Assessment of Included Studies.

Author/Year	Study Type	Cases (Number)	Groups	Interventions	Selection Bias (0–4)	Comparability (0–2)	Outcome Assessment (0–3)	Total NOS Score (0–9)	Risk of Bias
Salamun et al., 2018, [28]	RCT, Open-label, PCOS	28	MET vs. MET + Liraglutide	Liraglutide 1.2 mg + MET	3/4	2/2	3/3	8/9	Low
Liu et al., 2017, [29]	RCT, Open-label, PCOS	176	Exenatide vs. Metformin	Exenatide 10 μg BID	3/4	2/2	3/3	8/9	Low
Nylander et al., 2017, [30]	RCT, Double-blind, PCOS	72	Liraglutide vs. Placebo	Liraglutide 1.8 mg/day	4/4	2/2	3/3	9/9	Low
Sun et al., 2020, [18]	In vivo (PCOS mouse model) + In vitro granulosa cells	Not applicable (mouse + cell lines)	GLP-1RA-treated vs. untreated	GLP-1 (dose-dependent)	2/4	1/2	3/3	6/9	Moderate
Nishiyama et al., 2017, [31]	In vitro rat granulosa cells	Cell lines	Incretins (GLP-1, GIP) ± FSH, BMP-6	GLP-1/GIP stimulation	2/4	1/2	3/3	6/9	Moderate
Saber et al., 2019, [32]	In vivo animal study (Female rats)	30 rats	Control vs. Liraglutide vs. Recovery	Liraglutide (therapeutic dose)	2/4	1/2	3/3	6/9	Moderate

Randomised clinical trials, in vitro studies assessing the impact of GLP-1 receptor agonists on reproductive or ovarian endpoints, observational human studies, and experimental animal studies were all considered eligible. Menstrual regularity, ovulation, metabolic and androgen parameters, ovarian morphology, granulosa-cell function, expression of steroidogenic enzymes, oxidative stress, apoptosis, natural pregnancy, and IVF-related parameters were all considered eligible outcomes. Reviews, editorials, conference abstracts without complete data, non-reproductive studies, and publications written in languages other than English were all excluded. Table 1 delineates the criteria for inclusion and exclusion. Abbreviations: RCT, randomized controlled trial; PCOS, polycystic ovary syndrome; MET, metformin; GLP-1RA, glucagon-like peptide-1 receptor agonist; BID, twice daily; QD, once daily; SC, subcutaneous; NOS, Newcastle–Ottawa Scale; BMI, body mass index; DM, diabetes mellitus; MEN 2, multiple endocrine neoplasia type 2.

**Table 2:** Characteristics of Included Studies.

Year	Author	Country	Type of Study	Sample Size (Cases/Controls)	GLP-1RA Used	GLP-1RA Duration	Inclusion Criteria	Exclusion Criteria	Recruitment Period	IVF Protocol Used	Outcome Investigated	Results	Adverse Effects of GLP-1RA
**2018**	**Salamun et al.,** [28]	Slovenia	RCT	27 13 allocated to combination of metformin with liraglutide 14 allocated to only metformin	Liraglutide 1.2 mg QD SC	12 weeks 4-week medication-free period prior to IVF protocol	Women with PCOS BMI ≥ 30 kg/m^2^ ≤38 years old First or second IVF attempt	Severe male infertility DM Type I or II History of malignancy Personal or family history of MEN 2 Significant medical conditions Use of medications known to affect reproductive or metabolic functions Use of statins No ovarian pathology	2014–2015	Short GnRH antagonist protocol (cetrorelix)	Pregnancy Rate	Significantly higher pregnancy rates in the combination group compared to the metformin alone group	Nausea Headache
**2017**	**Nylander et al.,** [30]	Denmark	RCT	65 44 allocated to liraglutide 21 allocated to placebo	Liraglutide SC 1st week:0.6mg/day 2nd week: 1.2 mg/day 3rd–24th week: 1.8 mg/day	24 weeks	Women with PCOS BMI ≥ 25 kg/m^2^ >18 years old Insulin resistance	Known pregnancy DM Use of hormonal contraceptives Use of anti-diabetic medication Use of anti-androgenic medication Other causes of irregular menstruation	2014–2015	n/a	Bleeding ratio Ovarian volume stromal volume AFC serum levels of AMH	Significant difference in bleeding ratio Non-significant changes in ovarian volume, stromal volume, AFC and AMH	Nausea Constipation
**2019**	**Saber et al.,** [32]	Egypt	Animal study	30 10 allocated to placebo (Group 1) 10 allocated to liraglutide (Group 2) Liraglutide Group + 2 weeks of recovery period	Liraglutide SC 1st week: 0.6 mg/mL 2nd week: 1.2 mg/mly 3rd week: 1.8 mg/mL 4th week: 2.4 mg/mL	4 weeks	n/a	n/a	NR	n/a	LH, FSH, ER, PR, T levels Effect on granullosa ovarian cells Effect on endometrium	Significant decrease in FSH, LH, ER, PR and T levels Apoptosis of granullosa cells of follicles Inflammatory infiltration of endometrium	n/a
**2020**	**Sun et al.,** [18]	China	Animal study	50 10 (control group) 40 injected for 20 days with DHEA 6 mg/100 g/day followed by the injection of liraglutide	Liraglutide BID SC (0.2 mg/kg)	21 days	n/a	n/a	NR	n/a	Effect on Granullosa Cells Proliferation	Liraglutide demonstrated an improvement of granullosa cells proliferation Liraglutide facilitated follicular development and anti-apoptosis	n/a
**2018**	**Nishiyama et al.,** [31]	Japan	Animal study	Rats exposed to DES (10 mg) and FSH (30 ng/mL)	GLP-1RA (100 Nm)	n/a	n/a	n/a	NR	n/a	Effect on ovarian steroidogenesis in rat granullosa cells	Suppression of FSH-induced PR production after GLP-1RA Suppression of FSH- induced c AMP production after GLP-1RA Downregulation of the steroidogenic enzymes StAR, P450scc and 3β-HSD	n/a
**2017**	**Liu et al.,** [29]	China	RCT	176 88 allocated to exenatide 88 allocated to metformin	Exenatide BID (10 μg)	12 weeks	18–40 years old BMI ≥ 24 kg/m^2^ No use of contraception Presence of at least one normal fallopian tube Normal uterine cavity	Partner with abnormal sperm parameters DM Type I or II Significant medical conditions History of malignancy Use of medications known to affect reproductive or metabolic functions	NR	n/a	Effect on menstrual frequency ratio Effect on natural pregnancy rates	Significant improvement of menstrual frequency ratio of exenatide group Significantly increased rate of natural pregnancy in the exenatide group	Nausea Bloating Vomiting Dizziness Rash at the injection site

Table 2 outlines the essential elements of each trial in our analysis, including the research design, sample size, GLP-1RA agent and dosage, treatment duration, inclusion criteria, reproductive endpoints, and primary outcomes. Abbreviations: RCT, randomized controlled trial; GLP-1RA, glucagon-like peptide-1 receptor agonist; PCOS, polycystic ovary syndrome; BMI, body mass index; IVF, in vitro fertilization; GnRH, gonadotropin-releasing hormone; AFC, antral follicle count; AMH, anti-Müllerian hormone; LH, luteinizing hormone; FSH, follicle-stimulating hormone; ER, estrogen receptor; PR, progesterone receptor; T, testosterone; SC, subcutaneous; BID, twice daily; QD, once daily; NR, not reported; DM, diabetes mellitus; MEN 2, multiple endocrine neoplasia type 2.

**Table 3:** Summary of Main Findings Across Clinical, Animal, and In vitro Studies.

Evidence Level	Key Findings	Interpretation	Overall Direction
Clinical Studies (Human)	• GLP-1RAs improved menstrual regularity, ovulation, and natural pregnancy rates in overweight/obese women with PCOS. • Liraglutide + metformin increased IVF pregnancy rate (85.7% vs. 28.6%). • Significant improvements in weight, insulin resistance, SHBG, and free testosterone.	GLP-1RAs appear to enhance reproductive outcomes primarily in metabolically dysregulated populations (PCOS + obesity) through systemic metabolic correction and improved endocrine balance.	Beneficial for metabolic PCOS, improved fertility outcomes.
Animal Studies (In vivo)	• In PCOS-like models: GLP-1RA exposure increased granulosa-cell proliferation, reduced apoptosis, activated *FOXO1* phosphorylation, and improved ovarian health. • In healthy rodents: high-dose liraglutide caused granulosa-cell apoptosis, follicular atresia, oxidative stress, reduced FSH/LH/E2/PRG, and increased testosterone. • Partial recovery after drug discontinuation.	GLP-1RA actions are highly context-dependent. Beneficial in insulin-resistant ovaries but harmful in healthy ovaries under supraphysiological exposure. Highlights the importance of metabolic status and treatment duration.	Mixed (beneficial in PCOS-like models, potentially harmful in normal ovaries).
In vitro Studies	• GLP-1 increased granulosa-cell survival by inducing *FOXO1* phosphorylation and nuclear exclusion. • Modulated FSH–cAMP steroidogenesis by suppressing StAR, P450scc, and 3β-HSD. • Selective interaction with BMP–Smad signaling (GIP > GLP-1). • Receptor-dependent changes in ALK-3/ALK-6 and Smad6 expression.	GLP-1R signaling intersects with granulosa-cell survival, steroidogenesis, and intra-ovarian growth-factor pathways, offering mechanistic explanations for clinical endocrine improvements.	Mechanistically supportive of clinical benefits in PCOS.

A review of the principal results from in vitro, animal, and clinical research assessing the impact of GLP-1RAs in reproductive physiology. Clinical research consistently indicates that women with PCOS have enhanced hormonal, metabolic, and reproductive consequences. In vitro mechanistic data substantiate the direct influence of GLP-1 signalling on granulosa cell functionality and steroidogenesis. Animal studies reveal context-dependent effects, showing advantageous results in models with impaired metabolism and possible detrimental effects in healthy ovarian tissue when exposed to high dosages. Abbreviations: GLP-1RA, glucagon-like peptide-1 receptor agonist; PCOS, polycystic ovary syndrome; IVF, in vitro fertilization; FOXO1, forkhead box O1; BMP, bone morphogenetic protein; SHBG, sex hormone-binding globulin; FSH, follicle-stimulating hormone; LH, luteinizing hormone; E2, estradiol; PRG, progesterone; StAR, steroidogenic acute regulatory protein; cAMP, cyclic adenosine monophosphate.

## Data Availability

No new data were created or analyzed in this study. Data sharing is not applicable to this article.

## Abbreviations

GLP-1	glucagon-like peptide-1;
GLP-1R	glucagon-like peptide-1 receptor;
GLP-1RA	glucagon-like peptide-1 receptor agonist;
PCOS	polycystic ovary syndrome;
IVF	in vitro fertilization;
ART	assisted reproductive technology;
BMI	body mass index;
FSH	follicle-stimulating hormone;
LH	luteinizing hormone;
GnRH	gonadotropin-releasing hormone;
SHBG	sex hormone–binding globulin;
AMH	anti-Müllerian hormone;
E2	estradiol;
PRG	progesterone;
T	testosterone;
GC	granulosa cell;
FOXO1	forkhead box O1;
BMP	bone morphogenetic protein;
StAR	steroidogenic acute regulatory protein;
cAMP	cyclic adenosine monophosphate;
PKA	protein kinase A;
EPAC	exchange protein directly activated by cAMP;
PI3K	phosphoinositide 3-kinase;
AKT	protein kinase B;
HPO axis	hypothalamic–pituitary–ovarian axis;
AFC	antral follicle count;
NOS	Newcastle–Ottawa Scale;
RCT	randomized controlled trial;
SC	subcutaneous;
QD	once daily;
BID	twice daily;
NR	not reported;
DM	diabetes mellitus;
MEN 2	multiple endocrine neoplasia type 2.
